# Dual Targeted Nanoparticles for the Codelivery of Doxorubicin and siRNA Cocktails to Overcome Ovarian Cancer Stem Cells

**DOI:** 10.3390/ijms241411575

**Published:** 2023-07-18

**Authors:** Li Chen, Jinlan Luo, Jingyuan Zhang, Siyuan Wang, Yang Sun, Qinying Liu, Cui Cheng

**Affiliations:** 1 Fujian Provincial Key Laboratory of Tumor Biotherapy, Fujian Medical University Cancer Hospital & Fujian Cancer Hospital, Fuzhou 350014, China; ibptcl@fzu.edu.cn (L.C.); sunyang@fjmu.edu.cn (Y.S.); 2 Fujian Provincial Key Laboratory of Medical Instrument and Pharmaceutical Technology, College of Biological Science and Technology, Fuzhou University, Fuzhou 350108, China; ibptljl@163.com (J.L.); ibptzjy@126.com (J.Z.); ibptwsy@163.com (S.W.); 3 Department of Gynecology, Fujian Medical University Cancer Hospital & Fujian Cancer Hospital, Fuzhou 350014, China; 4 School of Basic Medical Sciences, Fujian Medical University, Fuzhou 350122, China; 5 College of Chemistry, Fuzhou University, Fuzhou 350108, China

**Keywords:** iRGD peptides, siRNAs, cancer stem cells, reduction sensitive, targeted delivery

## Abstract

Most anticancer treatments only induce the death of ordinary cancer cells, while cancer stem cells (CSCs) in the quiescent phase of cell division are difficult to kill, which eventually leads to cancer drug resistance, metastasis, and relapse. Therefore, CSCs are also important in targeted cancer therapy. Herein, we developed dual-targeted and glutathione (GSH)-responsive novel nanoparticles (SSBPEI–DOX@siRNAs/iRGD–PEG–HA) to efficiently and specifically deliver both doxorubicin and small interfering RNA cocktails (siRNAs) (survivin siRNA, Bcl-2 siRNA and ABCG2 siRNA) to ovarian CSCs. They are fabricated via electrostatic assembly of anionic siRNAs and cationic disulfide bond crosslinking-branched polyethyleneimine-doxorubicin (SSBPEI–DOX) as a core. Interestingly, the SSBPEI–DOX could be degraded into low-cytotoxic polyethyleneimine (PEI). Because of the enrichment of glutathione reductase in the tumor microenvironment, the disulfide bond (–SS–) in SSBPEI–DOX can be specifically reduced to promote the controlled release of siRNA and doxorubicin (DOX) in the CSCs. siRNA cocktails could specifically silence three key genes in CSCs, which, in combination with the traditional chemotherapy drug DOX, induces apoptosis or necrosis of CSCs. iRGD peptides and “sheddable” hyaluronic acid (HA) wrapped around the core could mediate CSC targeting by binding with neuropilin-1 (NRP1) and CD44 to enhance delivery. In summary, the multifunctional delivery system SSBPEI–DOX@siRNAs/iRGD–PEG–HA nanoparticles displays excellent biocompatibility, accurate CSC-targeting ability, and powerful anti-CSC ability, which demonstrates its potential value in future treatments to overcome ovarian cancer metastasis and relapse. To support this work, as exhaustive search was conducted for the literature on nanoparticle drug delivery research conducted in the last 17 years (2007–2023) using PubMed, Web of Science, and Google Scholar.

## 1. Introduction

Cancer stem cells (CSCs) represent a small population of tumor cells with strong self-renewal and continuous proliferation ability [1]. They are considered the root cause of tumor metastasis and recurrence and are characterized by active DNA repair ability, high expression of ABC (ATP-binding cassette) transporters, and anti-apoptosis properties. Targeting CSCs might be the key to overcoming malignancy. Unfortunately, conventional chemotherapeutic drugs or nucleic acid drugs may show therapeutic effects on rapidly growing tumors, but they may not be able to eliminate CSCs completely [2]. However, combination therapy might be a practical strategy to enhance the anti-CSC therapeutic effect.

CSCs are highly resistant to chemotherapy, which is a major problem when treating cancer patients, and CSCs survive with higher invasiveness [3,4]. Gene therapy is expected to intervene in the control of CSCs by specifically blocking signaling both in vitro and in vivo, silencing abnormal CSC-associated genes via miRNAs (micro RNAs) and siRNAs (small interfering RNAs), inhibiting CSC proliferation, and boosting apoptosis in CSCs [5,6,7,8]. However, it also has disadvantages, such as small cell intake, easy degradation, and poor tissue specificity [9,10,11].

In our study, we designed SSBPEI–DOX@siRNAs/iRGD–PEG–HA as a novel nanoparticle to overcome CSCs by integrating multiple properties and functions together, including good biocompatibility and targeting, tumor microenvironment responsiveness, high transport efficiency, and controlled release of loaded drugs in nanostructures.

Firstly, we specifically silenced three key genes in CSCs, survivin, Bcl-2, and ABCG2, and combined with the traditional chemotherapy drug doxorubicin (DOX) as the core of the nanomaterials. Survivin, Bcl-2, and ABCG2 are considered to be highly related to self-renewal differentiation, antiapoptosis, and drug resistance characteristics of CSCs, respectively. Secondly, the disulfide bond crosslinking-branched polyethyleneimine-doxorubicin (SSBPEI–DOX) could be degraded into low-cytotoxic polyethyleneimine (PEI) and the DOX could be released via a disulfide bond break in the presence of endogenous enzymes such as glutathione reductase [12,13,14]. Then, SSBPEI–DOX@siRNAs as a core, was obtained by loading siRNAs with electrostatic interactions. Thirdly, to improve the stability and specificity of the nano-delivery system, tumor targeting iRGD peptides, hyaluronic acid (HA) and polyethylene glycol (PEG) were introduced to SSBPEI–DOX@siRNA by electrostatic interaction, thereby creating self-assembled SSBPEI–DOX@siRNAs/iRGD–PEG–HA nanoparticles. The modification of mPEG on the surface of nanoparticles can prolong the circulation time of nanoparticles in vivo [15]. CD44, one of the most common markers of CSCs, can mediate the adhesion between cells and the extracellular matrix and participate in cell proliferation, differentiation, and adhesion migration [16,17,18]. CD44 may provide a new molecular target for cancer therapy and may also serve as a marker of poor prognosis in the cancer population [19]. HA, a major component of the extracellular matrix, has attracted widespread interest due to its excellent ability to target CD44 receptors on the surface of CSCs [20,21,22]. To strengthen the CSC-targeting specificity, we added the iRGD polypeptide to our nanosystems. The iRGD polypeptides can be cleaved by cell surface-associated proteases, which exposes a CendR motif (CRGDK) that binds neuropilin-1 (NRP1) while promoting cellular internalization [23,24,25]. Neuropilin-1 (NRP1) has been reported to be expressed by cancer stem-like cells and to be involved in cell spreading [26,27]. However, due to the heterogenicity and complexity of cancer, the therapeutic effectiveness of single-receptor-targeting nanomedicine is, unfortunately, limited. Compared to single-targeting, a dual-targeted, combination therapy allows for faster tumour capture and faster enrichment on the tumour surface, significantly facilitating drug enrichment at the tumour site; therefore, the concept of dual-receptor-targeted nanomedicine is an emerging trend driving the field of cancer treatment research [28,29,30].

In brief, our nanoparticles have DOX and siRNA loading capacity, reduction responsiveness, and CSC-targeting ability, which can provide a novel and effective drug delivery method for the future treatment of recurrent tumors.

## 2. Results

### 2.1. Synthesis and Characterization of SSBPEI–DOX@siRNAs/iRGD–PEG–HA Nanoparticles

**Scheme 1:**
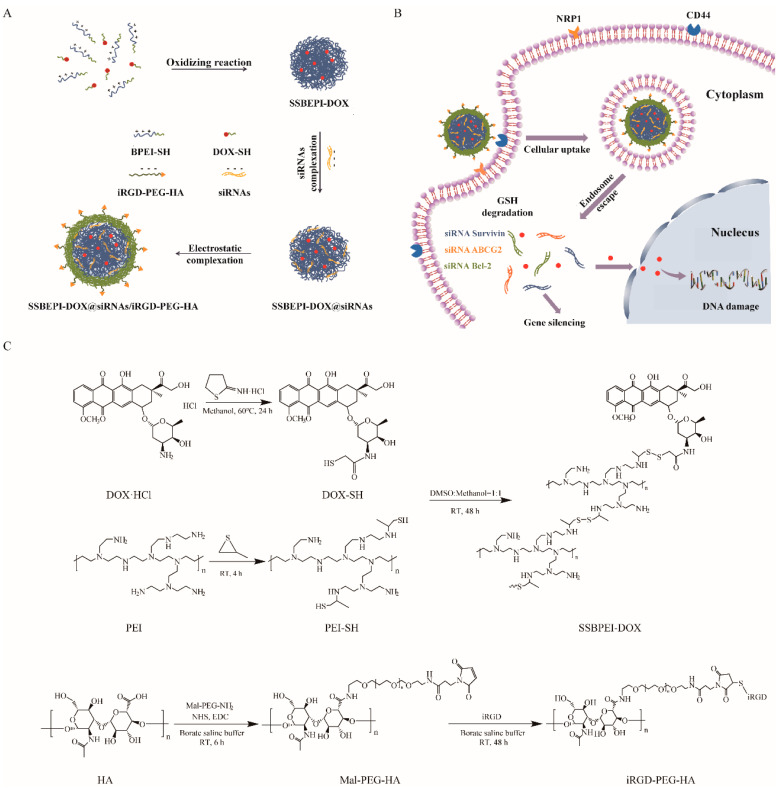
Schematic representation of (**A**) SSBPEI–DOX@siRNAs/iRGD–PEG–HA and (**B**) targeted delivery of SSBPEI–DOX@siRNAs/iRGD–PEG–HA nanoparticles. (**C**) Synthesis of SSBPEI–DOX and iRGD–PEG–HA.

Sulfhydrylated branched polyethylenimine (BPEI–SH) was synthesized by introducing the thiol moiety of propylene sulfide on the amine group of branched polyethyleneimine (BPEI1.2K). Sulfhydrylated doxorubicin (DOX–SH) was prepared by a ring-opening reaction of 2-iminothio ring hydrochloride (Trauťs reagent) with the amine group of doxorubicin hydrochloride (DOX·HCl) (Scheme 1C). Subsequently, sulfhydrylated polyethylenimine (PEI–SH) and DOX–SH were mixed to obtain SSBPEI–DOX through an oxidation reaction (Scheme 1C). In addition, Cys–iRGD and hyaluronic acid (HA) were attached to Mal-PEG-NH2 (Scheme 1C). Finally, as shown in Scheme 1A, disulfide bond crosslinking-branched polyethyleneimine-doxorubicin (SSBPEI–DOX) and siRNA were complexed. Then, iRGD-PEG-HA was added to obtain SSBPEI–DOX@siRNAs/iRGD–PEG–HA nanoparticles.

**Figure 1:**
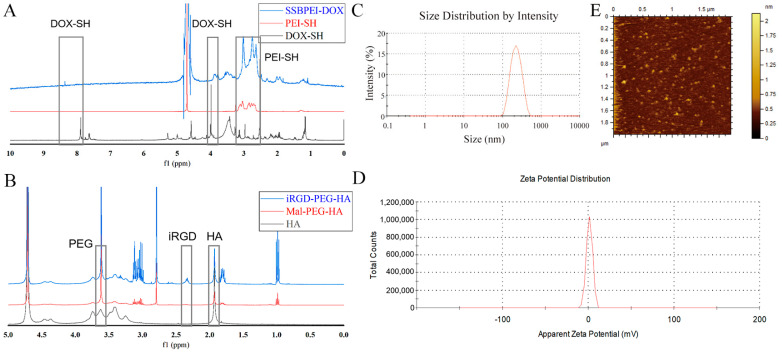
Characteristics of the polymers and nanoparticles. ^1^H NMR spectra of (**A**) SSBPEI–DOX and (**B**) iRGD–PEG–HA. (**C**) Size distribution and (**D**) zeta potential of SSBPEI–DOX@siRNAs/iRGD–PEG–HA (W/W = 1.5:1). (**E**) Morphology of SSBPEI–DOX@siRNAs/iRGD–PEG–HA nanoparticles observed by AFM.

The chemical structures of SSBPEI-DOX and iRGD-PEG-HA were confirmed by ^1^H-NMR. It can be seen that the spectrum of SSBPEI-DOX showed the characteristic peaks of –CH_2_–N at δ 2.5–3.0 ppm from PEI-SH, and the characteristic peaks of aromatic hydrogens at δ 8.3 ppm and –OCH_3_ at δ 3.9 ppm from DOX-SH, as shown in Figure 1A. Furthermore, the characteristic peaks of CH_3_–CO– at δ 1.9 ppm from HA, –OCH_2_– at δ 3.6 ppm from PEG, and –CH_2_– at δ 2.3 ppm from iRGD indicated the successful synthesis of iRGD-PEG-HA, as shown in Figure 1B.

Since the size and zeta potential of nanoparticles are critical for antitumor effects, we determined the particle size and zeta potential of SSBPEI–DOX@siRNAs/iRGD–PEG–HA nanoparticles by DLS, and further observed their morphology by AFM. As shown in Tables S1 and S2, the size of SSBPEI–DOX@siRNAs nanoparticles decreased (from 285.1 nm to 251.8 nm) with the increased ratio of the total number of nitrogen atoms in SSBPEI to the total number of phosphorus atoms in siRNA (N/P). At the same time, the surface charge increased with the increase of N/P (zeta potential increased from −6.8 mV to 30.3 mV). Based on the data above, we selected SSBPEI–DOX@siRNAs nanoparticles with an N/P ratio of 5:1 to further explore. At the same time, with the increase of the weight ratio of @siRNAs, the zeta potential decreased from +24.7 mV to −13.8 mV, and the size decreased from 307.5 nm to 189.7 nm (Table S3). As a result, we finally identified SSBPEI–DOX@siRNAs/iRGD–PEG–HA nanoparticles with a mass ratio of 1.5:1 for the follow-up study. As shown in Figure 1C,D, the SSBPEI–DOX@siRNAs/iRGD–PEG–HA nanoparticles with a mass ratio of 1.5:1 exhibited an average particle size of 225 nm and a surface charge of 4.1 mV, indicating a well-dispersed particle size distribution and zeta potential distribution. Moreover, as the AFM image was shown (Figure 1E), the nanoparticles were uniformly distributed in spherical structures.

### 2.2. Extraordinary Stability and Reduction Sensitivity of SSBPEI–DOX@siRNAs/iRGD–PEG–HA Nanoparticles

It is well known that siRNAs could lose their mobility in electric fields, when complexed with cationic polymers through electrostatic interactions. Therefore, we performed a gel retardation assay to measure the binding capacity of polymers to siRNAs. The results showed the encapsulation ability of SSBPEI–DOX was enhanced with the increase of N/P ratio (Figure S1A), and complete complexation of siRNA could be achieved when the N/P ratio was greater than or equal to 5:1 (Figure S1B). Additionally, complete blockade of siRNA migration was induced whether nanoparticles were coated with iRGD–PEG–HA or not, indicating complete neutralization of siRNA negative charges. In summary, the SSBPEI–DOX@siRNAs/iRGD–PEG–HA nanoparticles could achieve stable loading of siRNAs.

Disulfide bonds linking the drug to the carrier in SSBPEI–DOX could be reduced in the glutathione-rich tumor microenvironment. Since the disruption of disulfide bonds favors the release of DOX and siRNAs from nanoparticles, we monitored the size change of DDT-treated nanoparticles by DLS with DTT-untreated nanoparticles as a control. As shown in Figure S1C, the average size of the DTT-treated nanoparticles increased dramatically, while the control group remained largely unchanged, indicating a significant structural transformation of the nanoparticles. Simultaneous TEM images also showed changes in the nanoparticle structure (Figure S1D).

### 2.3. Validation of Biological Characteristics of Stem Cell Microspheres

**Figure 2:**
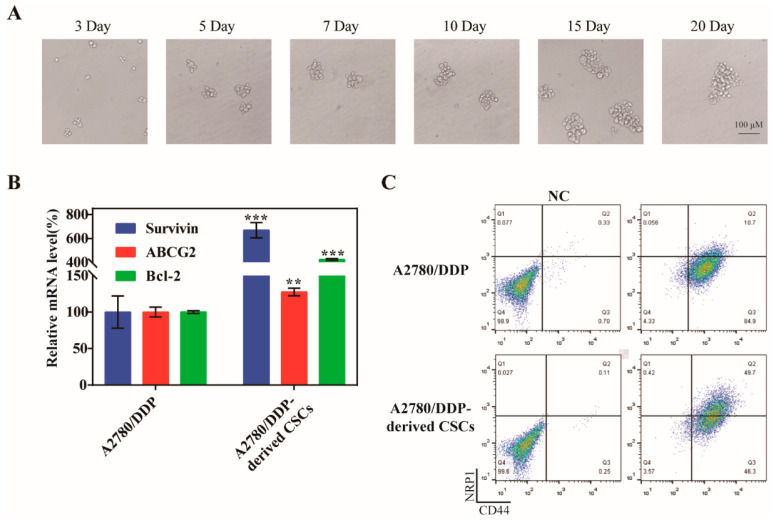
Culture of A2780/DDP-derived CSCs. (**A**) Representative images of spheroids formed from A2780/DDP cells at days 3, 5, 7, 10, 15, and 20 (upper panel). Scale bar = 100 µm. (**B**) qPCR analysis of survivin, ABCG2, and Bcl-2 in A2780 parent cells and A2780/DDP-derived CSCs. The mRNA levels of each gene were quantified and normalized to those of U6. (**C**) Flow cytometry analysis of CD44 and NRP1 expression in A2780/DDP parent cells and A2780/DDP-derived CSCs. The percentages of the CD44+ NRP1+ population are indicated. The results are reported as the mean ± SD (*n* = 3). ** *p* < 0.01, *** *p* < 0.001.

Numerous of studies have shown that drug resistance and CSCs are inextricably linked, precisely because some characteristics of CSCs can accumulate mutations caused by chemotherapy drugs and pass them down from generation to generation. To obtain CSCs, we cultured the drug-resistant ovarian cancer cells A2780/DDP under serum-free culture conditions and changed the medium every three days. The tumor microspheres gradually formed on the fifth day, and the size of the microspheres tended to stabilize after 15 days (Figure 2A). We also measured the mRNA levels of survivin, Bcl-2, and ABCG2 in both A2780/DDP parent cells and A2780/DDP-derived CSCs, and found that the expression of the three genes was greater in the CSCs compared with the parent cells (Figure 2B). The expression of the CSC biomarkers CD44 and NRP1 on the cell surface of A2780/DDP cells was greater after microsphere culture (Figure 2C), which was more beneficial to the active targeting of SSBPEI–DOX@siRNAs/iRGD–PEG–HA.

### 2.4. Dual Targeting Ability of the Nanoparticles In Vitro/Vivo

**Figure 3:**
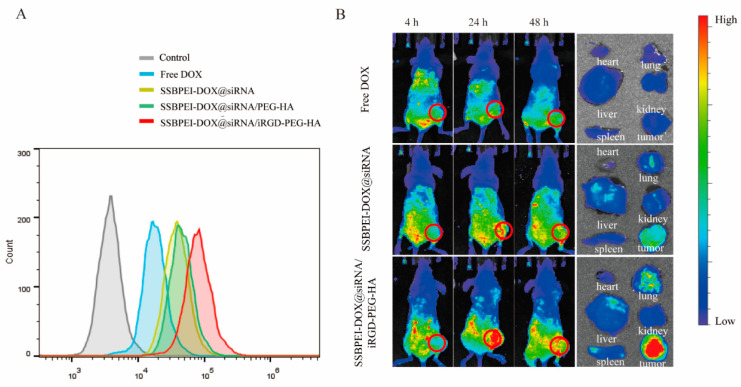
Dual targeting ability of the nanoparticles in vitro and in vivo. (**A**) FACS analysis of A2780/DDP-derived CSCs treated with free DOX, SSBPEI–DOX@siRNAs, SSBPEI–DOX@siRNAs/mPEG–HA, or SSBPEI–DOX@siRNAs/iRGD–PEG–HA. (**B**) In vivo NIRF images of the nude mice bearing A2780/DDP-derived CSC tumors at 4, 24, and 48 h postinjection and ex vivo NIRF images of major organs and tumors extracted from the mice injected with free DOX, SSBPEI–DOX@siRNAs, and SSBPEI–DOX@siRNAs/iRGD–PEG–HA serial nanoparticles.

To verify that SSBPEI–DOX@siRNAs/iRGD–PEG–HA can specifically target NRP-1 and CD44 receptors on the CSC surface, we analyzed the uptake of nanoparticles in CSCs by flow cytometry. As shown in Figure 3A, A2780/DDP-derived CSCs had low uptake of free DOX. In contrast, higher fluorescence intensities were detected in A2780/DDP-derived CSCs incubated with nanoparticles of different compositions. Particularly, the A2780/DDP-derived CSCs cocultured with the nanoparticles coating of iRGD and HA showed the strongest fluorescence intensity (red curve). These results suggested that SSBPEI–DOX@siRNAs/iRGD–PEG–HA could specifically target to A2780/DDP-derived CSCs, whose membranes are rich in NRP1 and CD44.

To further test the tumor-targeting properties of SSBPEI–DOX@siRNAs/iRGD–PEG–HA nanoparticles, we examined the biodistribution of DOX-loaded nanoparticles in mice. The dual-targeted modification of iRGD and HA on the complex significantly increased DOX enrichment at tumor sites (Figure 3B). There was no obvious DOX enrichment in the tumor site after 24 h of free DOX intravenous injection. Conversely, the SSBPEI–DOX@siRNAs/iRGD–PEG–HA group showed distinct DOX signals. This could be attributed to the active targeting properties of the iRGD– and HA–modified complexes, which interacted with their corresponding cell surface receptors NRP1 and CD44, respectively. This action promotes nanocomplex binding to tumor cells and receptor-mediated endocytosis. In addition, the signal intensity of the SSBPEI–DOX@siRNAs/iRGD–PEG–HA group was still strong 48 h after injection, indicating that the gene-drug complex was efficiently accumulated in the tumor. The partial tumor accumulation of SSBPEI–DOX@siRNAs was estimated using passive targeting, mediated by EPR. Mice were sacrificed 24 h later and tumors and major organs were subjected to fluorescence imaging. As shown in Figure 3B, the free DOX and SSBPEI–DOX@siRNAs were distributed mainly in the liver and kidneys, while the SSBPEI–DOX@siRNAs/iRGD–PEG–HA complex was distributed primarily in the tumor site. Taken together, the cross-linking strategy enhanced drug accumulation at the tumor site.

Hence, the SSBPEI–DOX@siRNAs/iRGD–PEG–HA nanoparticles exhibited excellent targeting ability in vitro and in vivo due to the dual-targeting modification, and may be used for synergistic targeted drug delivery.

### 2.5. The Efficiency of Nanoparticle-Silencing of Survivin, Bcl-2, and ABCG2 Genes

**Figure 4:**
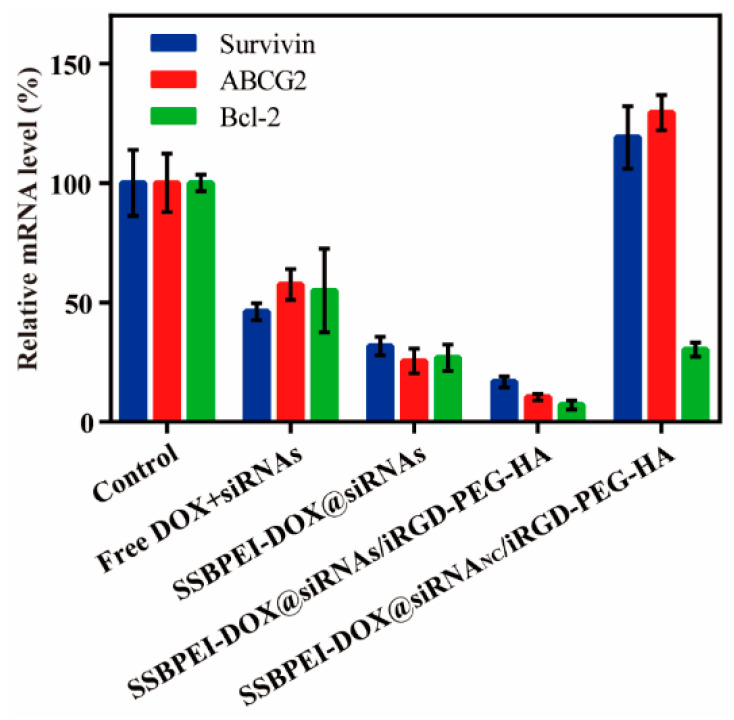
The mRNA levels of survivin, ABCG2 and Bcl-2 in A2780/DDP-derived CSCs treated with free DOX, SSBPEI–DOX@siRNAs, SSBPEI–DOX@siRNAs/mPEG–HA or SSBPEI–DOX@siRNAs/iRGD–PEG–HA were detected by qPCR. The results are reported as the mean ± SD (*n* = 3).

The free siRNAs are very limited in gene-silencing due to its instability; therefore, suitable carriers are needed to improve the stability and deliver efficiency of siRNAs. Our nanoparticles were developed to silence survivin, Bcl-2, and ABCG2 in A2780/DDP-derived CSCs, and the gene-silencing efficiencies of different components were evaluated by qRT-PCR. Our results showed that the gene expression levels of survivin, Bcl-2, and ABCG2 were downregulated by varying degrees in A2780/DDP-derived CSCs treated with SSBPEI–DOX@siRNAs, SSBPEI–DOX@siRNAs/PEG–HA, and SSBPEI–DOX@siRNAs/iRGD–PEG–HA nanoparticles. Moreover, SSBPEI–DOX@siRNAs/iRGD–PEG–HA nanoparticles showed the most significant regulation, indicating that iRGD– and HA–mediated endocytosis of nanoparticles can effectively silence specific gene expression. Surprisingly, the expression of survivin and ABCG2 in A2780/DDP-derived CSCs treated with SSBPEI–DOX@siRNANC/iRGD–PEG–HA nanoparticles was also slightly upregulated, which appears to be the normal response of CSCs to DOX stimulation, phenotypically causing resistance to apoptosis and promotion of drug resistance through related gene regulation. It should be noted that the expression of these three genes in A2780/DDP-derived CSCs treated with free siRNAs were down-regulated as well, although not as effectively. We assumed that free siRNAs might be taken up by cells in a passive manner (Figure 4).

### 2.6. The Anti-CSC Effect of the Nanoparticles In Vitro

**Figure 5:**
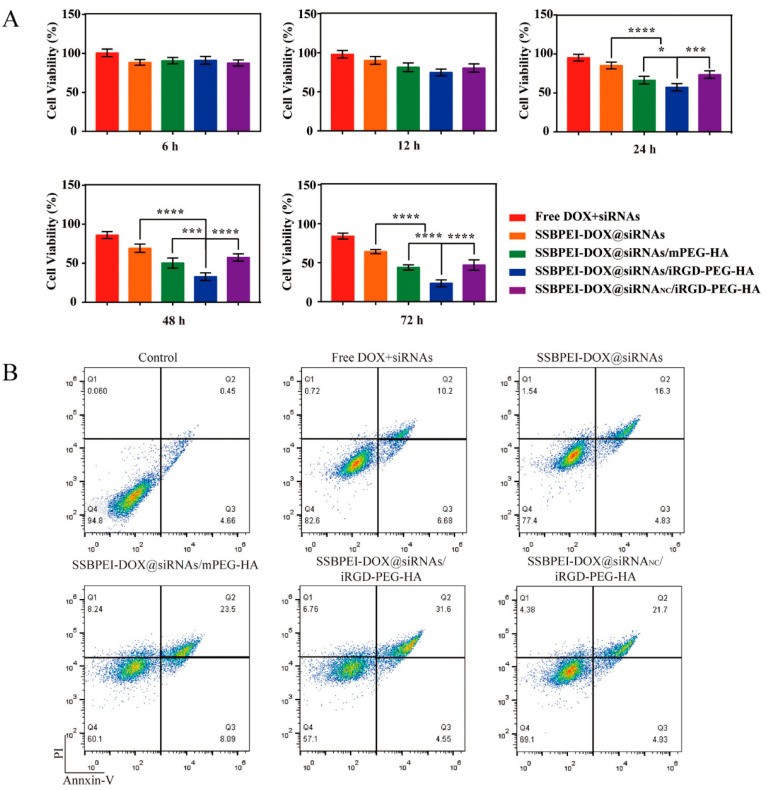
The anti-CSC effect of the nanoparticles. (**A**) The viability of A2780/DDP-derived CSCs treated with free DOX, SSBPEI–DOX@siRNAs, SSBPEI–DOX@siRNAs/mPEG–HA, SSBPEI–DOX@siRNAs/iRGD–PEG–HA or SSBPEI–DOX@siRNA_NC_/iRGD–PEG–HA for 6, 12, 24, 48 and 72 h. (**B**) Annexin V-FITC/PI test of apoptosis in A2780/DDP-derived CSCs incubated with free DOX, SSBPEI–DOX@siRNAs, SSBPEI–DOX@siRNAs/mPEG–HA, SSBPEI–DOX@siRNAs/iRGD–PEG–HA or SSBPEI–DOX@siRNA_NC_/iRGD–PEG–HA for 72 h. The results are reported as the mean ± SD (*n* = 3). * *p* < 0.05, *** *p* < 0.001, **** *p* < 0.0001.

Downregulation of survivin, Bcl-2, and ABCG2 is known to be an effective means of promoting apoptosis in CSCs. Thus, after exposing CSCs to the different drug formulations mentioned above, for different durations, we evaluated the level of cell viability by CCK-8. As shown in Figure 5A, after 6 h of co-incubation, there was no significant difference in the effect of the materials on cell viability among the groups. But the cell viability of A2780/DDP-derived CSCs treated with SSBPEI–DOX@siRNAs/iRGD–PEG–HA was significantly lower than the other groups, and was reduced to a minimum of 23.73 ± 4.71% after 72 h treatment. We further analyzed the apoptosis of A2780/DDP-derived CSCs after 72 h treatment with different drug preparations, using Annexin V-FITC/PI double staining, as shown in Figure 5B. The results were consistent with the results obtained from CCK-8 assay; SSBPEI–DOX@siRNAs/iRGD–PEG–HA effectively induced apoptosis of A2780/DDP-derived CSCs, indicating active targeting of iRGD and HA to CSCs and effective synergistic activity of the combination of DOX and related gene (survivin, Bcl-2, and ABCG2) silencing.

**Figure 6:**
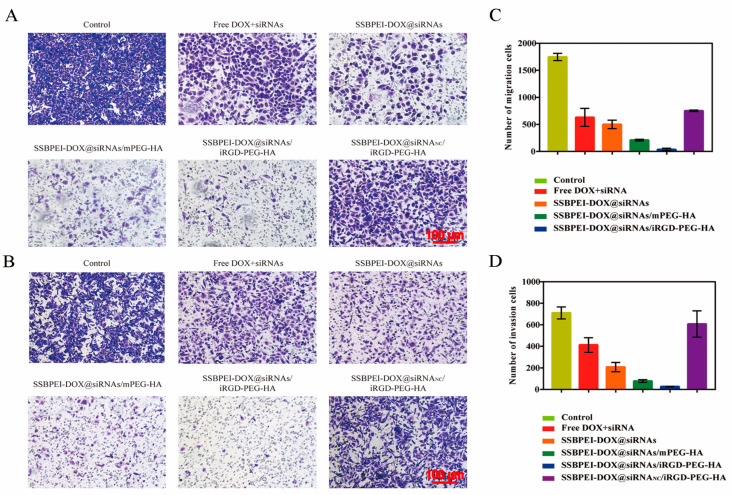
In vitro cell migration and invasion. (**A**) Transwell migration (scale: 100 μm) and (**B**) invasion images of A2780/DDP-derived CSCs treated with free DOX, SSBPEI–DOX@siRNAs, SSBPEI–DOX@siRNAs/mPEG–HA, SSBPEI–DOX@siRNAs/iRGD–PEG–HA or SSBPEI–DOX@siRNA_NC_/iRGD–PEG–HA for 48 h (scale: 100 μm). The numbers of (**C**) migratory and (**D**) invasive cells were calculated. The results are reported as the mean ± SD (*n* = 3).

The antimetastatic effect of SSBPEI–DOX@siRNAs/iRGD–PEG–HA on A2780/DDP-derived CSCs was evaluated by cell migration and invasion assessment. As shown in Figure 6A–D, the control group had the most effective migration and invasion, due to the nonfunction of siRNANC, followed by the SSBPEI–DOX@siRNANC/iRGD–PEG–HA group. The SSBPEI–DOX@siRNAs and SSBPEI–DOX@siRNAs/mPEG–HA groups displayed a smaller number of migrating and invading cells because SSBPEI-DOX@siRNAs and SSBPEI–DOX@siRNAs/mPEG–HA could deliver some siRNAs into A2780/DDP-derived CSCs, which led to some antimetastatic and anti-invasion effects. As expected, the SSBPEI–DOX@siRNAs/iRGD–PEG–HA group exhibited the lowest levels of migrating and invading cells, indicating that the dual-targeting and dual-drug-loading structure can deliver DOX and siRNAs to A2780/DDP-derived CSCs, specifically and can effectively inhibit migration and invasion.

### 2.7. Hemocompatibility of the Nanoparticles

**Figure 7:**
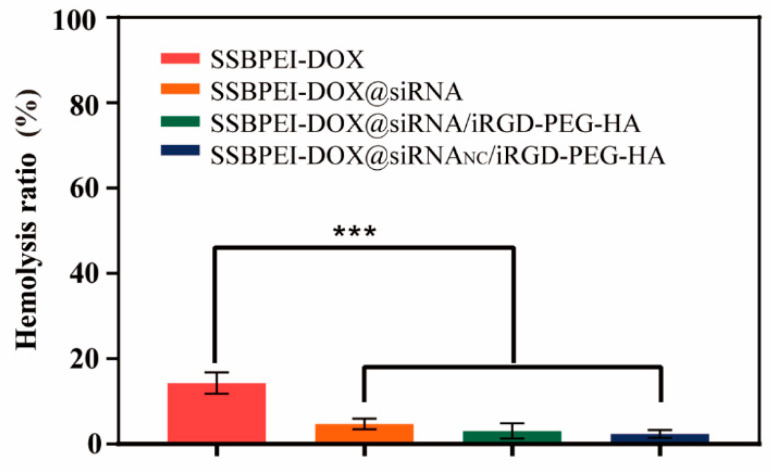
Hemolysis ratio of SSBPEI–DOX, SSBPEI–DOX@siRNAs, SSBPEI–DOX@siRNAs/PEG–HA or SSBPEI–DOX@siRNAs/iRGD–PEG–HA. The results are reported as the mean ± SD (*n* = 3). *** *p* < 0.001.

The hemolysis rate is used to measure the extent of damage to the erythrocyte membrane when in contact with various nanoparticles. A hemolysis rate less than 5% usually indicates that the nanoparticles have good blood compatibility. As shown in Figure 7, hemolysis of SSBPEI–DOX@siRNAs/iRGD–PEG–HA nanoparticles (under 5%) is less than that of SSBPEI–DOX (17%). This is due to the strong electropositivity of SSBPEI–DOX, which has a large destructive capacity for the negative biofilm. With the addition of negatively charged iRGD–PEG–HA, the final nanoparticles have the lowest hemolysis rate (less than 3%), confirming the good biocompatibility of the nanoparticles.

## 3. Discussion

In this study, with goals of active targeting and efficient delivery, a drug/gene nano-delivery system with tumor microenvironment responsiveness and CSC-specificity targeting was designed and constructed: SSBPEI–DOX@siRNAs/iRGD–PEG–HA. The synergistic effect, resulting from the efficient uptake of DOX and various siRNAs (siRNA survivin, siRNA ABCG2, and siRNA Bcl-2), improved the antitumor efficacy. iRGD has demonstrated superior capabilities for efficient tumor targeting and tumor penetration [31]. The iRGD–PEG–HA shell was shown to specifically target CSCs with high expression of CD44 and NRP1. Moreover, a high level of GSH in tumor cells and the microenvironment led to the removal of the nanoparticle shell and efficient release of DOX and siRNAs. Compared to free DOX, the iRGD–PEG–HA modified nanoparticles showed excellent ability to promote drug uptake by CSCs, which depended on the active targeting properties against marker molecules CD44 and NRP1 on the surface of CSCs, rather than the EPR effect. In addition, the nanoparticles combined with the chemotherapeutic drug DOX and the cancer gene-silencing drug siRNAs showed acceptable stability and gene-silencing efficiency, which is amplified by the double-targeted modification of HA and iRGD. More importantly, it greatly improves the killing and invasion-inhibiting abilities of A2780/DDP-derived CSCs. This finding is consistent with several other reports. Wang et al. demonstrated that survivin downregulation combined with a low dose of doxorubicin inhibited stemness and eliminated CSCs in mice bearing chemoresistant tumors [32]. In another study, a PEI-based NP system was synthesized to codeliver doxorubicin, aspirin, and Bcl-2 siRNA to colorectal and breast cancer cells. This strategy reduces the resistance of colon and breast cancer cells to chemotherapeutic drugs by delivering Bcl-2 siRNA and DOX simultaneously [33]. These results indicated that SSBPEI–DOX@siRNAs/iRGD–PEG–HA nanoparticles may be a promising nanocarrier for the coordinated therapy of chemotherapy and cancer gene-silencing in cancer treatment [34]. At the same time, polyethyleneimine (PEI) is a green biomaterial with good biocompatibility and safety. Ahmadov et al. designed a simple, green, and low-cost AgNP, using the aqueous extracts of roots as the reducing agent [35]. Hasanzadeh et al. investigated an eco-friendly, and highly efficient Fe_3_O_4_ magnetic nanoparticle for targeted delivery of DOX [36]. A study of mitochondria-targeting magnetic composite nanoparticles (MMCNs), has shown that composite nanoparticles can selectively target cancer cells and deliver to mitochondria [37]. With the development of biomedical materials, green biomaterials are gaining more and more attention. Fortunately, SSBPEI–DOX@siRNAs/iRGD–PEG–HA nanoparticles are designed to combine the advantages of eco-friendliness, efficient dual-targeting, good biocompatibility, and low toxicity.

## 4. Materials and Methods

### 4.1. Materials

Branched polyethylenimine (BPEI, MW = 1200, BPEI_1.2K_) was purchased from Alfa Aesar (Shanghai, China). MAL–PEG–NH_2_ (MW = 2000) was purchased from Yare Biotech Inc. (Shanghai, China). Doxorubicin hydrochloride (DOX·HCl) and cisplatin (DDP) was purchased from Meilun Biotechnology Co. (Dalian, China). Dihydrothiophen-2(3H)-imine hydrochloride (2-IT) was purchased from Macklin (Shanghai, China). HA (MW = 50,000) was purchased from Sai Taisi Biotechnology Co. (Nanjing, China). iRGD peptides, siRNAs were purchased from Sangon Biotech Co. (Shanghai, China). The siRNAs were double-stranded and more stable in the external environment. Agarose and ethidium bromide was purchased from Beyotime (Shanghai, China).

### 4.2. Synthesis of BPEI–SH and DOX–SH

The synthetic method of BPEI–SH is consistent with our previous work [38,39]. Briefly, BPEI-SH was obtained by reacting BPEI_1.2K_ with propylene sulfide under nitrogen protection stirred at 60–70 °C for 18–24 h.

For the synthesis of DOX–SH, DOX·HCl (10 mg) was dissolved in EDTA (1 mM) mixed solution. At a molar ratio of 1:5, 2-IT was added and stirred for 4 h at RT under nitrogen protection in the dark. Subsequently, the solution was dialyzed in ultrapure water replaced daily and lyophilized to obtain DOX-SH.

For the synthesis of SSBPEI–DOX, BPEI–SH (1 g) was dissolved in DMSO. DOX–SH was added with a sulfur content ratio of 1:3 and stirred at RT for 48 h. Subsequently, the solution was dialyzed in ultrapure water replaced daily and lyophilized to obtain SSBPEI–DOX.

### 4.3. Synthesis of iRGD–PEG–HA

For the synthesis of iRGD–PEG–HA, HA (100 mg), NHS (89.2 mg), EDC (145.6 mg) and MAL–PEG–NH_2_ (15.5 mg) were dissolved in ultrapure water. The mixture was stirred for 6 h at RT, iRGD (8.8 mg) was added, and the mixture was stirred for another 48 h. Subsequently, the solution was dialyzed in ultrapure water replaced daily and lyophilized to obtain white floc iRGD–PEG–HA.

### 4.4. Preparation of SSBPEI–DOX@siRNAs and SSBPEI–DOX@siRNAs/iRGD–PEG–HA Nanoparticles

SSBPEI–DOX and siRNAs were dissolved in enzyme-free water, respectively, and SSBPEI–DOX@siRNAs nanoparticles with different nitrogen-to-phosphorus ratios (N/P) were formed by electrostatic interaction. The nitrogen content of the SSBPEI was measured by an organic elemental analyzer and the phosphorus content of the siRNA was calculated. The ratio of the nitrogen content of the SSBPEI to the phosphorus content of the siRNA as N/P. Subsequently, iRGD–PEG–HA was added to the systems and further incubated at 4 °C for more 15 min to obtain SSBPEI–DOX@siRNAs/iRGD–PEG–HA nanoparticles with different weight ratios (*w*/*w*).

### 4.5. Characterization of Nanoparticles

Structures of SSBPEI–DOX and iRGD–PEG–HA confirmed by proton nuclear magnetic resonance (^1^H NMR, D_2_O, Bruker, Karlsruhe, Germany). The particle size, potential and polydispersity index (PDI) of SSBPEI–DOX@siRNA, SSBPEI–DOX@siRNAs/mPEG–HA and SSBPEI–DOX@siRNAs/iRGD–PEG–HA nanoparticles dissolved in 1.2 mL ultrapure water were determined by dynamic light scattering (DLS; Zetasizer Nano-ZS, Malvern Instruments Ltd., UK). The morphology of the SSBPEI–DOX@siRNAs/iRGD–PEG–HA nanoparticles (W/W = 1.5:1) were characterized by atomic force microscopy (AFM, JPK NanoWizard II instrument, Karlstuhe, Germany).

### 4.6. Gel Retardation Assay

The nanoparticles should have good siRNA encapsulation ability, and the gel retardation test can be used to determine the complexation ability of the polymer to siRNA. Thus, agarose gel electrophoresis was performed after mixing the above nanoparticles with loading buffer (6×) in different ratios (1% TAE agarose). Nucleic acid bands with ethidium bromide (EB) were visualized by UV (wavelength: 302 nm).

### 4.7. Reduction Responsiveness Test

The reduction responsiveness of SSBPEI–DOX@siRNAs/iRGD–PEG–HA nanoparticles was evaluated by changes in particle size and morphology. SSBPEI–DOX@siRNAs/iRGD–PEG–HA (W/W = 1.5:1) were incubated with 15 mM DTT for 4 h at 37 °C and measured by DLS. Then, the morphology of the nanoparticles was visualized by TEM (TECNAI G2 F20, FEI, Salem, OR, USA), while the nanoparticles without DTT treatment were set as a control.

### 4.8. Hemolysis Assay

Fresh blood from New Zealand rabbits was collected and red blood cells (RBCs) were isolated. Mix the samples with 2% RBCs and incubate for 2 h at 37 °C. The mixture was then centrifuged and the supernatant was analyzed to determine the rate of hemolysis. Hemolysis rate determined by testing the amount of haemoglobin released.

### 4.9. Cell Culture

#### 4.9.1. A2780/DDP Cell Culture

The human drug-resistant ovarian cancer cell line (A2780/DDP) was purchased from the Shanghai Cell Resource Center (Shanghai, China). Cells were cultured separately in RPMI 1640 (Hyclone, Logan, UT, USA) medium supplemented with 10% (*v*/*v*) fetal bovine serum (Gemini, Woodland, CA, USA), 2 mmol/L DDP and 1% (*v*/*v*) dual antibodies.

#### 4.9.2. Spherical Culture of Stem Cell Microspheres

Low melting point agarose and DMEM/F12 (1:1) (Hyclone, Logan, UT, USA) medium were mixed to form a 4% agarose solution and sterilized at 121 °C for 25 min. An appropriate amount of agarose solution was added to the cell culture dish while it hot, and it was left to stand in the ultraclean workbench for 30 min to obtain a low cell adhesion culture flask.

A2780/DDP cells in the logarithmic growth phase were harvested and resuspended at appropriate density in DMEM/F12 culture medium supplemented with 20 ng/mL EGF, 20 ng/mL bFGF and 2% B27 (Gibco, Carlsbad, CA, USA)). The cell suspension was dispersed in an ultralow adhesion culture dish and placed in a cell culture incubator with medium changes every three days. The samples were centrifuged to collect microspheres, the dead cells and single cells were removed, and fresh stem cell culture medium was added.

### 4.10. Characterization of Sphere-Forming Cells

The expression levels of CD44 and NRP1 on the surface of A2780/DDP cells and A2780/DDP-derived CSCs were evaluated by fluorescence-activated cell sorting (FACS). In brief, A2780/DDP-derived CSCs were harvested and resuspended in PBS with 3% FBS. The cells were incubated with anti-CD44 (Clone IM7; BD Bioscience; America) and anti-NRP1 (Clone U21-1283; BD Bioscience; San Jose, CA, USA) antibodies, and incubated 30 min at RT in dark. The samples were analyzed by flow cytometry (BD FACS Verese, San Jose, CA, USA) after washing and resuspension.

### 4.11. Detection of Cell Uptake by Flow Cytometry

Cellular internalization was evaluated by FACS. Nanoparticles were co-cultured with A2780/DDP-derived CSCs for an additional 4 h at 37 °C. Subsequently, cells were harvested and resuspended in PBS with 3% FBS before analysis by flow cytometry.

### 4.12. In Vivo Biodistribution

The study was approved by the Ethics Committee of Fuzhou University. BALB/cJ nude mice were 5–6 weeks old and purchased from Guangzhou Yaokang. All experiments with animals took place at the Fuzhou University. All experiments were carried out in accordance with the Fuzhou University guidelines for the welfare of experimental animals.

First, 5 × 10^5^ A2780/DDP cells were injected subcutaneously into the right hindlimb of mice. After 14 days of culture, the tumor size was approximately 150–200 mm^3^. Mice were randomized into 3 groups (*n* = 3) for in vivo biodistribution studies. Mice were administered free DOX, SSBPEI–DOX@siRNAs or SSBPEI–DOX@siRNAs/iRGD–PEG–HA nanoparticles respectively by i.v. injection. Drug distribution behavior was monitored using the Caliper IVIS Lumina XRMS in vivo imaging system, and images were acquired at 4-, 24-, and 48-h post-injection. After 24 h, the tumors and main organs were removed for fluorescence imaging.

### 4.13. Real-Time PCR

The levels of survivin, Bcl-2 and ABCG2 were quantified by qPCR. Total RNA was isolated from A2780/DDP and A2780/DDP-derived CSCs using TRIzol reagent, and cDNA was obtained Total RNA was isolated from A2780/DDP and A2780/DDP-derived CSCs using TRIzol reagent, and cDNA was obtained by reverse transcription from mRNA followed by quantitative PCR. U6 small nuclear RNA was selected as the internal control (U6 primer: Forward: 5′-CTCGCTTCGGCAGCACA-3′; Reverse: 5′-AACGCTTCACGAATTTGCGT-3′). Then, A2780/DDP-derived CSCs were incubated with free DOX + siRNA mixture, SSBPEI–DOX@siRNAs (N/P = 5:1), SSBPEI–DOX@siRNAs/PEG–HA (W/W = 1.5:1), SSBPEI–DOX@siRNAs/iRGD–PEG–HA (W/W = 1.5:1) and SSBPEI–DOX@siRNANC/iRGD–PEG–HA (W/W = 1.5:1) for 72 h. The levels of surviving (surviving primer: Forward: 5′-CGCGGGACCCGTTGGCAGAG-3′; Reverse: 5′-GGAATTCGGCAGCTCCGGCCAGAGG-3′), Bcl-2 (Bcl-2 primer: Forward: 5′-GCATCTTCTCCTCGCAGCCC-3′; Reverse: 5′-CACCACCGTGGCAAAGCGTC-3′) and ABCG2 (ABCG2 primer: Forward: 5′-TTAGCTGCAAGGAAAGATCCAAG-3′; Reverse: 5′-GTCATAGTTGTTGGAAGCCGAAG-3′) in each group were quantified by the same method as above. Data analysis by 2^−ΔΔCt^ method.

### 4.14. Cytotoxicity

Cytotoxicity of nanoparticles against A2780/DDP-derived CSCs was evaluated using standard Cell Counting Kit-8 analysis. In brief, A2780/DDP-derived CSCs were mixed with the above-mentioned nanoparticles and plated in 96-well plates at the same density. After 6, 12, 24, 48, or 72 h of incubation, CCK-8 solution was added and incubated for 2 h, respectively. Finally, the optical density was evaluated at 450 nm. GraphPad Prism 7.0 software was used to calculate cell viability (%).

### 4.15. Annexin V-FITC/PI Double Staining

A2780/DDP-derived CSCs were incubated with different materials for 72 h, and the degree of apoptosis was detected by Annexin V-FITC/PI apoptosis detection kit. In brief, cells were harvested individually and resuspended in binding buffer. After the cell suspension was mixed and incubated with Annexin V-FITC and PI, the degree of apoptosis was detected by flow cytometry.

### 4.16. In Vitro Cell Migration and Invasion Assay

For the migration assay, A2780/DDP-derived CSCs were collected and resuspended in free DOX+siRNAs, SSBPEI–DOX@siRNAs (N/P = 5:1), SSBPEI–DOX@siRNAs/PEG–HA (W/W = 1.5:1), SSBPEI–DOX @siRNAs/iRGD–PEG–HA (W/W = 1.5:1) and SSBPEI–DOX@siRNANC/iRGD–PEG–HA (W/W = 1.5:1) containing serum-free medium. Untreated cells were used as controls. Then, the above cells were transferred to the upper chamber of Transwell. Meanwhile, cell migration was induced by adding medium containing 20% FBS to the lower chamber. After 24 h, cell migration was observed by microscopy after fixation with 4% paraformaldehyde and staining with crystal violet. The proportion of cell migration was calculated using Image J.

For the invasion assay, Matrigel was spread in the Transwell chamber, and the other steps of the invasion experiment were the same as those for migration.

### 4.17. Statistical Analysis

All data are shown as mean ± standard deviation (SD). Statistical comparisons between experimental groups were made using Student’s t test or analysis of variance (ANOVA). *p* values < 0.05 (*) were considered statistically significant, ** *p* < 0.01, *** *p* < 0.001.

## 5. Conclusions

In conclusion, we developed novel reduction-responsive dual-targeting DOX prodrug and siRNA coloaded nanoassemblies. These nanoassemblies, SSBPEI–DOX@siRNAs/iRGD–PEG–HA, showed high efficiency in CSC targeting and delivery by specifically binding with CD44 and NRP1 on the surface of CSCs. Moreover, they enhanced DOX cytotoxicity to CSCs through the simultaneous siRNA-silencing of the CSC-related genes survivin, Bcl-2 and ABCG2. As a result, SSBPEI–DOX@siRNA/iRGD–PEG–HA could greatly reduce tumor metastasis and invasion, leading to superior tumor regression. The nanoparticle could solve the problems of poor targeting and degradation by nucleases in the process of siRNA delivery in vivo, and have a better targeting and gene-silencing effect on tumor stem cells, providing a new idea for future gene therapy and tumor metastasis and recurrence due to CSCs.

## Data Availability

The datasets generated and analyzed during the present study are available from the corresponding author on reasonable request.

## Supplementary Materials

The following supporting information can be downloaded at: https://www.mdpi.com/article/10.3390/ijms241411575/s1.
