# Supplementary material for: Dual Targeted Nanoparticles for the Codelivery of Doxorubicin and siRNA Cocktails to Overcome Ovarian Cancer Stem Cells
Source: Int J Mol Sci. 2023 Jul 18;24(14):11575. doi: 10.3390/ijms241411575 (PMC10380749; doi:10.3390/ijms241411575)
Supplement: Supplementary file 1 [file ijms-24-11575-s001.zip › ijms-2494792-supplementary.pdf]

Supporting Information for: Dual targeted nanoparticles for the codelivery of doxorubicin and siRNA cocktails to overcome ovarian cancer stem cells

Li Chen , Jinlan Luo , Jingyuan Zhang , Siyuan Wang , Yang Sun , Qinying Liu [*]*, Cui Cheng* [*]

[*]Corresponding authors: liuqinying@fjmu.edu.cn (Q. Liu), ibptcc@fzu.edu.cn (C. Cheng)

Table S1

Contents of carbon, nitrogen, hydrogen and sulfur in BPEI-DOX、DOX-SH and SSBPEI-DOX.

Sample	Carbon content(%)	Nitrogen content (%)	Hydrogen content (%)	Sulfur content (%)
BPEI-SH	35.85	19.81	9.22	1.79
DOX-SH	16.15	1.81	1.48	2.90
SSBPEI-DOX	34.13	10.86	8.09	/

Table S2

Physicochemical property of SSBPEI-DOX@siRNA nanoparticles.

SSBPEI-DOX/ siRNA(N/P)	Size (nm)	Zeta potential (mV)	PDI
0.5:1	285.1±9.1	-6.8±1.7	0.21±0.02
1:1	255.8±3.2	-4.4±0.9	0.27±0.01
2:1	250.0±1.8	12.5±3.0	0.22±0.02
5:1	261.6±1.5	22.8±2.7	0.18±0.10
10:1	251.8±1.8	30.3±1.8	0.22±0.03

Measured in pH = 7.4 PBS. The results were reported as mean ± SD (n = 3).

Table S3

Physicochemical property of SSBPEI-DOX@siRNA nanoparticles.

BPEI-DOX@siRNA/ iRGD-mPEG-HA (W/W)	Size (nm)	Zeta potential (mV)	PDI
0.5:1	307.5±4.1	24.7±0.4	0.15±0.08
1:1	236.4±1.3	19.1±1.0	0.05±0.03
1.5:1	225.0±1.4	1.3±0.7	0.13±0.05
2:1	189.7±5.6	-13.8±0.7	0.16±0.06

Measured in pH = 7.4 PBS. The results were reported as mean ± SD (n = 3).

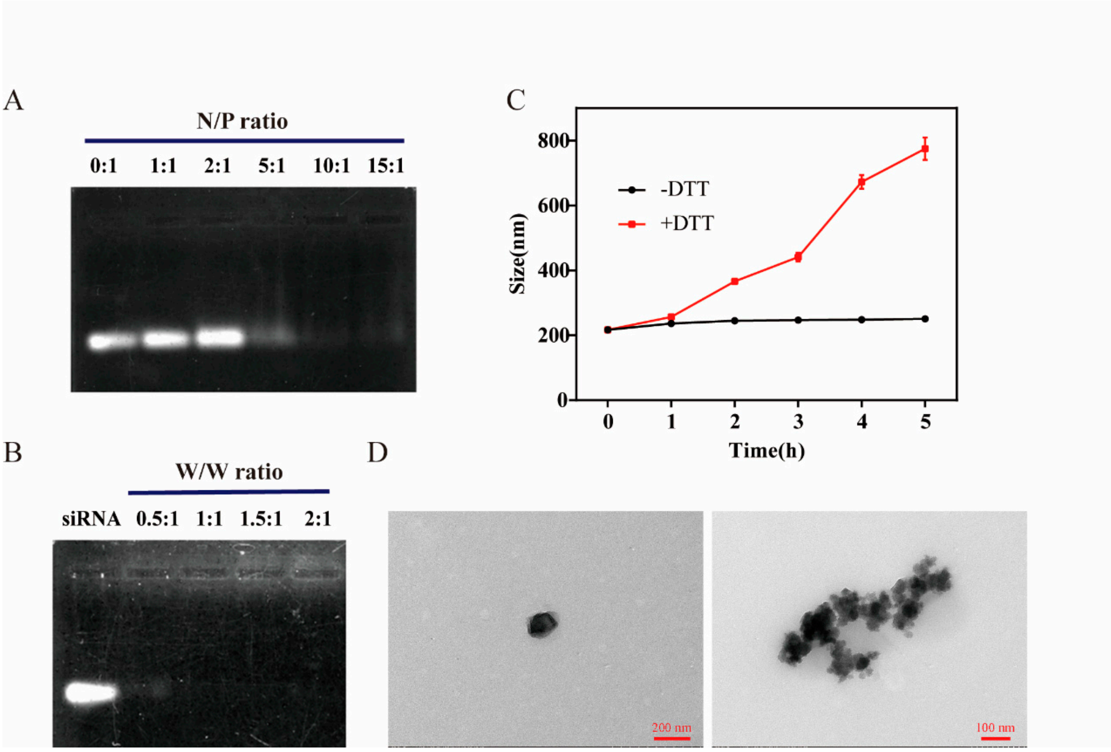

Figure S1. Gel retardation analysis of (A) SSBPEI-DOX@siRNAs and (B) SSBPEI-DOX@siRNAs/iRGD-PEG-HA; (C) Size distribution data and (D) TEM images of the SSBPEI-DOX@siRNAs/iRGD-PEG-HA nanoparticles with and without treatment with 15 mM DTT. The results were reported as mean ± SD (n = 3).
